# Supplementary material for: Singlet and triplet to doublet energy transfer: improving organic light-emitting diodes with radicals
Source: Nat Commun. 2022 May 18;13:2744. doi: 10.1038/s41467-022-29759-7 (PMC9117228; doi:10.1038/s41467-022-29759-7)
Supplement: Supplementary file 1 — Supplementary Information [file 41467_2022_29759_MOESM1_ESM.pdf]

Supplementary Information:
Singlet and triplet to doublet energy transfer: improving organic light-emitting diodes with radicals

Emrys W. Evans[2,3+], Alexander J. Gillett[2+], Qinying Gu[2], Junshuai Ding[1], Zhangwu Chen[1], Timothy J. H. Hele[4], William K. Myers[5], Richard H. Friend[2]*, Feng Li[1,2]*

[1]*State Key Laboratory of Supramolecular Structure and Materials, College of Chemistry, Jilin University, Qianjin Avenue 2699, Changchun, 130012, P. R. China*

[2]*Cavendish Laboratory, University of Cambridge, JJ Thomson Avenue, Cambridge, CB3 0HE, United Kingdom*

[3]*Department of Chemistry, Swansea University, Singleton Park, Swansea, SA2 8PP, United Kingdom*

[4]*Department of Chemistry, University College London, Christopher Ingold Building, London, WC1H 0AJ, United Kingdom*

[5]*Centre for Advanced Electron Spin Resonance (CAESR), Department of Chemistry, University of Oxford, Inorganic Chemistry Laboratory, South Parks Road, Oxford, OX1 3QR, United Kingdom*

[+]denotes equal contribution
*e-mail correspondence to: rhf10@cam.ac.uk (RHF), lifeng01@jlu.edu.cn (FL)

Contents

1. Theoretical considerations of energy transfer by FRET and Dexter mechanisms 2
The energy transfer system 2
Spin 3
Electronic interaction 4
2. Transient absorption (TA) studies of 4CzIPN and TTM-3PCz films 5
TA of 4CzIPN:CBP 5
TA of TTM-3PCz:CBP 6
TA of 4CzIPN:TTM-3PCz:CBP 7
3. Electroluminescence time dependence of 4CzIPN:TTM-3PCz:CBP OLEDs 8
4. Steady-state photoluminescence of 4CzIPN:TTM-3PCz:CBP film 9
5. Magneto-conductance studies of 4CzIPN:CBP and 4CzIPN:TTM-3PCz:CBP OLEDs 10
6. Temperature dependence on transient photoluminescence of 4CzIPN:CBP and 4CzIPN:TTM-3PCz:CBP films 11

1. Theoretical considerations of energy transfer by FRET and Dexter mechanisms

Here we consider the electronic structure algebra of the following energy transfer and emission processes:

$$\mathrm{T_1 + D_0 \rightarrow S_0 + D_1 \rightarrow S_0 + D_0} + h\nu. \qquad \text{Eq. 1a}$$

$$\mathrm{S_1 + D_0 \rightarrow S_0 + D_1 \rightarrow S_0 + D_0} + h\nu. \qquad \text{Eq. 1b}$$

In this section we demonstrate that both these processes are fully quantum mechanically spin-allowed (without requiring spin-orbit coupling) as they can conserve both total spin S and spin projection M_s.

The energy transfer system

We consider a minimal four (spatial) orbital, five-electron model that comprises: the HOMO of the radical energy acceptor, A (orbital 1); the SOMO of A (orbital 3); the HOMO of the energy donor, D (orbital 2); and the LUMO of D (orbital 4).

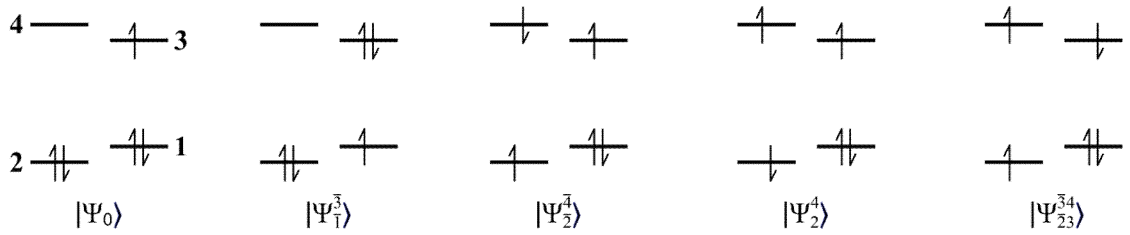

The electronic states considered are:

$$|\Psi_0\rangle = |1\bar{1}2\bar{2}3\rangle = |\mathrm{D(S_0)A(D_{0,+1/2})}\rangle \qquad \text{Eq. 2a}$$

$$\left|\Psi_{\bar{1}}^{\bar{3}}\right\rangle = |1\bar{3}2\bar{2}3\rangle = |\mathrm{D(S_0)A(D_{1,+1/2})}\rangle \qquad \text{Eq. 2b}$$

$$\left|\Psi_{\bar{2}}^{\bar{4}}\right\rangle = |1\bar{1}2\bar{4}3\rangle \qquad \text{Eq. 2c}$$

$$\left|\Psi_{2}^{4}\right\rangle = |1\bar{1}4\bar{2}3\rangle \qquad \text{Eq. 2d}$$

$$\left|\Psi_{\bar{2}3}^{\bar{3}4}\right\rangle = |1\bar{1}2\bar{3}4\rangle = |\mathrm{D(T_{1,+1})A(D_{0,-1/2})}\rangle \qquad \text{Eq. 2e}$$

where no bar indicates electron-spin 'up' and bar indicates electron-spin 'down'.

We wish to identify these determinants in the basis of the $\mathrm{S_0}$, $\mathrm{S_1}$ and $\mathrm{T_1}$ electronic states of D, and the $\mathrm{D_0}$ and $\mathrm{D_1}$ states of A. This is already set out Eq. 2a, Eq. 2b and Eq. 2e. This can be achieved from Eq. 2c and Eq. 2d by taking linear combinations:

$$\frac{1}{\sqrt{2}}\left(\left|\Psi_{\bar{2}}^{\bar{4}}\right\rangle + \left|\Psi_{2}^{4}\right\rangle\right) = |\mathrm{D(S_1)A(D_{0,+1/2})}\rangle \qquad \text{Eq. 3}$$

$$\frac{1}{\sqrt{2}}\left(\left|\Psi_{2}^{\bar{4}}\right\rangle - \left|\Psi_{2}^{4}\right\rangle\right) = |\mathrm{D}(\mathrm{T}_{1,0})\mathrm{A}(\mathrm{D}_{0,+1/2})\rangle. \qquad \text{Eq. 4}$$

For states of non-zero spin (doublets and triplets) we have added a subscript index of the projection spin quantum number, M_S.

Spin

All determinants in Eq. 2 are eigenstates of the $\hat{S}_z$ operator with eigenvalue, M_S = + $1/2$. In addition, $|\Psi_0\rangle$ and $\left|\Psi_{1}^{\bar{3}}\right\rangle$ are eigenstates of the $\hat{S}^2$ operator with eigenvalue: $3/4 = 1/2\,(1 + 1/2)$ and are doublet-spin states. However the other three determinants are not eigenstates of $\hat{S}^2$, and can be evaluated from three state basis in spin matrix:

$$\mathbf{S^2} \mapsto \begin{pmatrix} 7/4 & -1 & -1 \\ -1 & 7/4 & +1 \\ -1 & +1 & 7/4 \end{pmatrix}. \qquad \text{Eq. 5}$$

Eq. 5 has eigenvalues $15/4 = (3/2) \times (5/2)$, corresponding to a quartet, and $\frac{3}{4} = \frac{1}{2} \times \frac{3}{2}$ twice, corresponding to two doublets.

The quartet is uniquely defined as:

$$|\mathrm{Q}\rangle = \sqrt{\tfrac{2}{3}}\,|\mathrm{D}(\mathrm{T}_{1,0})\mathrm{A}(\mathrm{D}_{0,+1/2})\rangle - \sqrt{\tfrac{1}{3}}\,\left|\mathrm{D}(\mathrm{T}_{1,+1})\mathrm{A}(\mathrm{D}_{0,-\frac{1}{2}})\right\rangle \mapsto \sqrt{\tfrac{1}{3}}\begin{pmatrix} +1 \\ -1 \\ -1 \end{pmatrix}, \qquad \text{Eq. 6}$$

and the two doublet states can be chosen to be:

$$|\mathrm{D}(a)\rangle = \sqrt{\tfrac{1}{3}}\,|\mathrm{D}(\mathrm{T}_{1,0})\mathrm{A}(\mathrm{D}_{0,+1/2})\rangle + \sqrt{\tfrac{2}{3}}\,|\mathrm{D}(\mathrm{T}_{1,+1})\mathrm{A}(\mathrm{D}_{0,-1/2})\rangle \mapsto \sqrt{\tfrac{1}{6}}\begin{pmatrix} +1 \\ -1 \\ +2 \end{pmatrix} \qquad \text{Eq. 7}$$

$$|\mathrm{D}(b)\rangle = |\mathrm{D}(\mathrm{S}_1)\mathrm{A}(\mathrm{D}_{0,+1/2})\rangle \mapsto \sqrt{\tfrac{1}{2}}\begin{pmatrix} +1 \\ +1 \\ 0 \end{pmatrix}. \qquad \text{Eq. 8}$$

Note that $|\mathrm{D}(\mathrm{S}_1)\mathrm{A}(\mathrm{D}_{0,+1/2})\rangle$ is an eigenstate of $\hat{S}^2$ whereas $|\mathrm{D}(\mathrm{T}_{1,0})\mathrm{A}(\mathrm{D}_{0,+1/2})\rangle$ and $|\mathrm{D}(\mathrm{T}_{1,+1})\mathrm{A}(\mathrm{D}_{0,-1/2})\rangle$ are not. Although different linear combinations of the doublet eigenstates can be taken, it is possible to verify that the triplet-doublet determinants cannot be eigenstates of $\hat{S}^2$.

Electronic interaction

We aim to find the interaction between the spin eigenstates $|\mathrm{Q}\rangle$, $|\mathrm{D}(a)\rangle$, $|\mathrm{D}(b)\rangle$ and the emissive doublet state $\left|\Psi_{1}^{\bar{3}}\right\rangle = |1\bar{3}2\bar{2}3\rangle = |\mathrm{D}(\mathrm{S}_0)\mathrm{A}(\mathrm{D}_{1,+1/2})\rangle$. We find:

$$\left\langle\Psi_{1}^{\bar{3}}\middle|\hat{H}\middle|\Psi_{2}^{\bar{4}}\right\rangle = (13|24) - (12|34) \quad \text{Eq. 9}$$

$$\left\langle\Psi_{1}^{\bar{3}}\middle|\hat{H}\middle|\Psi_{2}^{4}\right\rangle = (13|24) \quad \text{Eq. 10}$$

$$\left\langle\Psi_{1}^{\bar{3}}\middle|\hat{H}\middle|\Psi_{23}^{\bar{3}4}\right\rangle = -(12|34) \quad \text{Eq. 11}$$

where we have integrated out the spin contributions and written the integrals in chemists' notation (Szabo A, Ostlund NS. *Modern quantum chemistry: introduction to advanced electronic structure theory*, 2012).

$$(12|34) = \int d\boldsymbol{r}_1 \int d\boldsymbol{r}_2 \psi_1^*(\boldsymbol{r}_1)\psi_2(\boldsymbol{r}_1)\frac{1}{r_{12}}\psi_3^*(\boldsymbol{r}_2)\psi_4(\boldsymbol{r}_2)$$

where $\psi_2(\boldsymbol{r}_1)$ corresponds to the value of the wavefunction of orbital 2 at position $\boldsymbol{r}_1$.

The integral $(13|24)$ is a dipole-dipole (FRET) term at lowest order, whereas $(12|34)$ is a Dexter electron-exchange term. From evaluating the $|\mathrm{Q}\rangle$, $|\mathrm{D}(a)\rangle$, $|\mathrm{D}(b)\rangle$ terms we find:

$$\left\langle\Psi_{1}^{\bar{3}}\middle|\hat{H}\middle|\mathrm{Q}\right\rangle = 0 \quad \text{Eq. 12}$$

$$\left\langle\Psi_{1}^{\bar{3}}\middle|\hat{H}\middle|\mathrm{D}(a)\right\rangle = -\sqrt{\frac{3}{2}}(12|34) \quad \text{Eq. 13}$$

$$\left\langle\Psi_{1}^{\bar{3}}\middle|\hat{H}\middle|\mathrm{D}(b)\right\rangle = \sqrt{2}(13|24) - \sqrt{\frac{1}{2}}(12|34) \quad \text{Eq. 14}$$

This means that $|\mathrm{D}(\mathrm{S}_1)\mathrm{A}(\mathrm{D}_{0,+1/2})\rangle$ and $|\mathrm{D}(\mathrm{S}_0)\mathrm{A}(\mathrm{D}_{1,+1/2})\rangle$ interact by both FRET and Dexter terms (with FRET term expected to dominate). There is no interaction between quartet state $|\mathrm{Q}\rangle$ and $|\mathrm{D}(\mathrm{S}_0)\mathrm{A}(\mathrm{D}_{1,+1/2})\rangle$, as expected on grounds of spin conservation, and the total triplet-doublet (D_1) pair interacts only via a Dexter-type term.

2. Transient absorption (TA) studies of 4CzIPN and TTM-3PCz films

TA of 4CzIPN:CBP

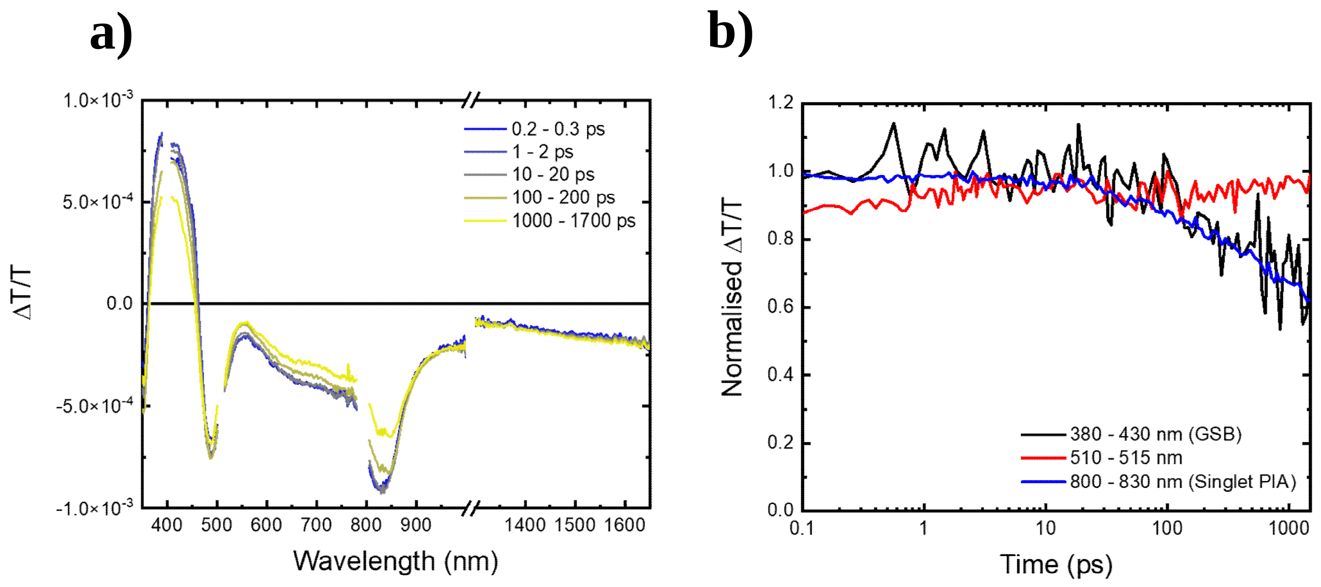

Supplementary Figure 1. Short-time TA of 4CzIPN:CBP with 400 nm excitation. a) Δ*T/T* timeslices of 4CzIPN:CBP (0.25:0.75) films with 400 nm excitation at 89.1 µJ/cm^2 fluence, from 0.2-0.3 ps to 1000-1700 ps. b) Normalised Δ*T/T* kinetic profiles of 380-430 nm (ground state bleach, GSB); 510-515 nm and 800-830 nm that are photoinduced absorption (PIA) signals we assign to 4CzIPN S_1. Discontinuities in timeslice spectral profiles for a) are due to multiple experiments used to cover the studied wavelength probe regions.

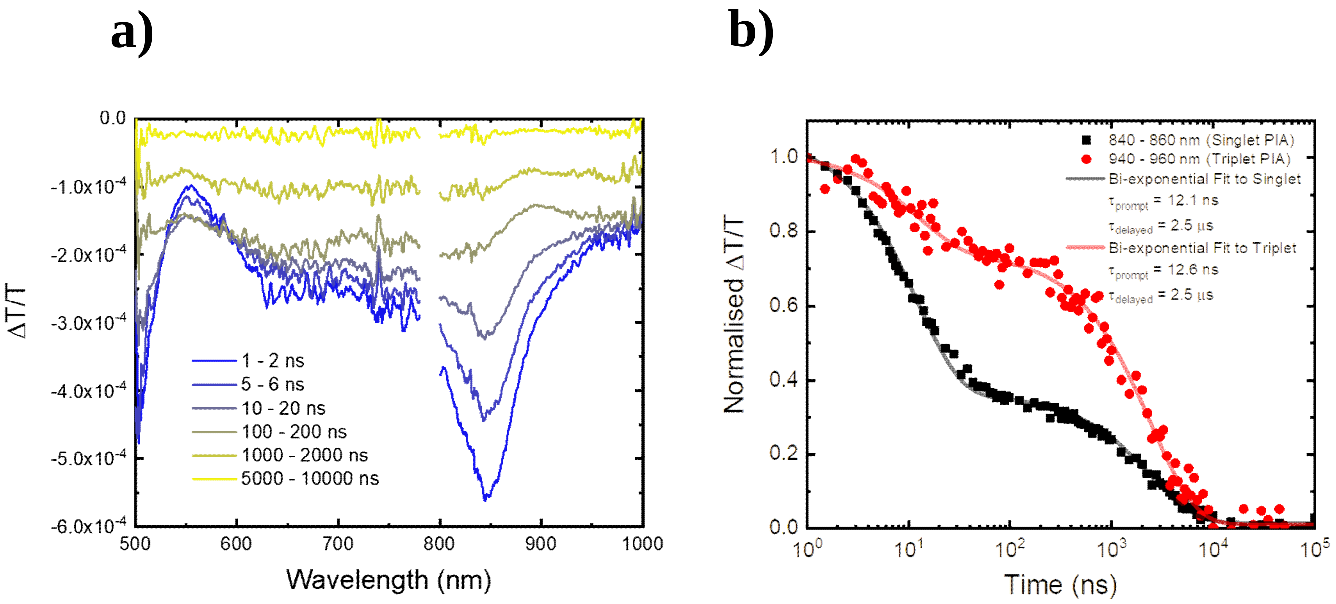

Supplementary Figure 2. Long-time TA of 4CzIPN:CBP with 355 nm excitation. a) Δ*T/T* timeslices of 4CzIPN:CBP (0.25:0.75) films with 355 nm excitation at 17.0 µJ/cm^2 fluence, from 1-2 ns to 5000-10000 ns. b) Normalised Δ*T/T* kinetic profiles of 840-860 nm (we assign to 4CzIPN S_1 PIA from Fig. S1a); and 940-960 nm (here we assign to 4CzIPN T_1 PIA). Discontinuities in timeslice spectral profiles for a) are due to multiple experiments used to cover the studied wavelength probe regions.

TA of TTM-3PCz:CBP

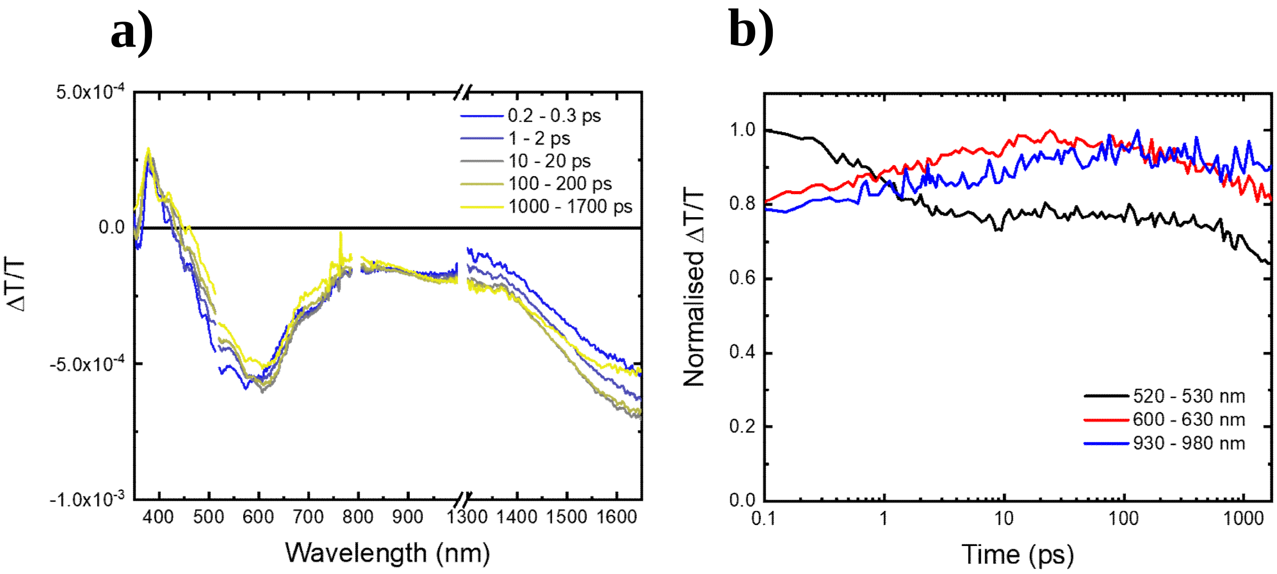

Supplementary Figure 3. Short-time TA of TTM-3PCz:CBP with 400 nm excitation. a) Δ*T/T* timeslices of TTM-3PCz:CBP (0.03:0.97) films with 400 nm excitation at 89.1 μJ/cm^2 fluence, from 0.2-0.3 ps to 1000-1700 ps. b) Normalised Δ*T/T* kinetic profiles of 520-530 nm; 600-630 nm and 930-980 nm that are photoinduced absorption (PIA) signals we assign to excited states of TTM-3PCz. Discontinuities in timeslice spectral profiles for a) are due to multiple experiments used to cover the studied wavelength probe regions.

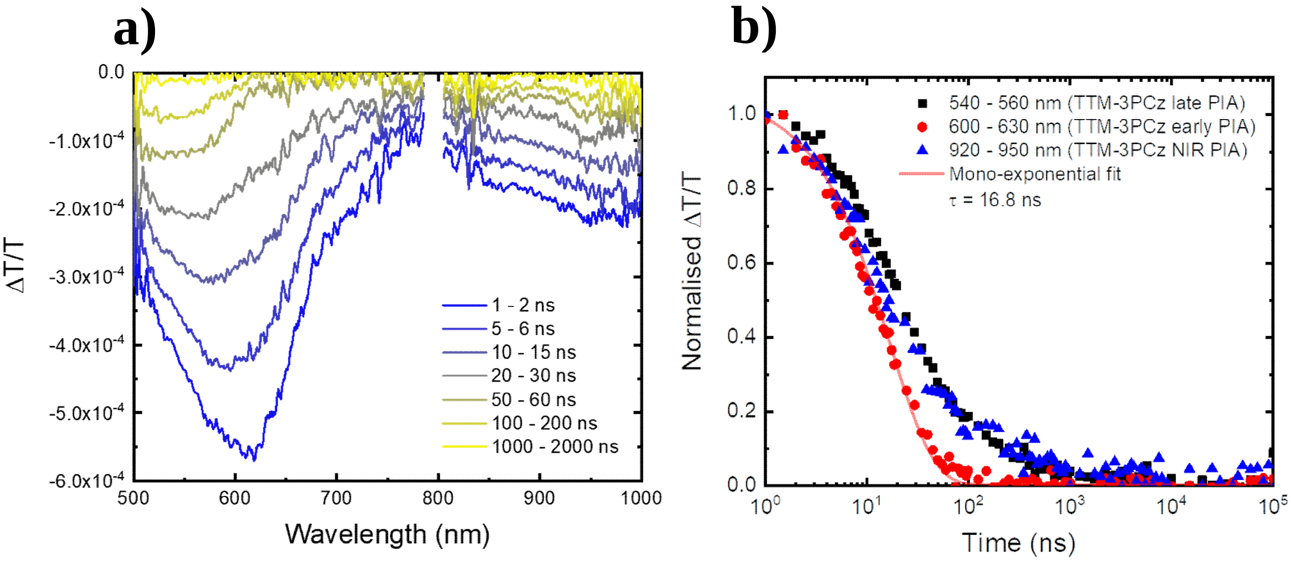

Supplementary Figure 4. Long-time TA of TTM-3PCz:CBP with 355 nm excitation. a) Δ*T/T* timeslices of TTM-3PCz:CBP (0.03:0.97) films with 355 nm excitation at 3.85 μJ/cm^2 fluence, from 1-2 ns to 1000-2000 ns. b) Normalised Δ*T/T* kinetic profiles of 540-560 nm (TTM-3PCz, late PIA from short-time TA); 600-630 nm (TTM-3PCz, early PIA from short-time TA) and 920-950 nm (TTM-3PCz, near-infrared wavelength PIA). Discontinuities in timeslice spectral profiles for a) are due to multiple experiments used to cover the studied wavelength probe regions.

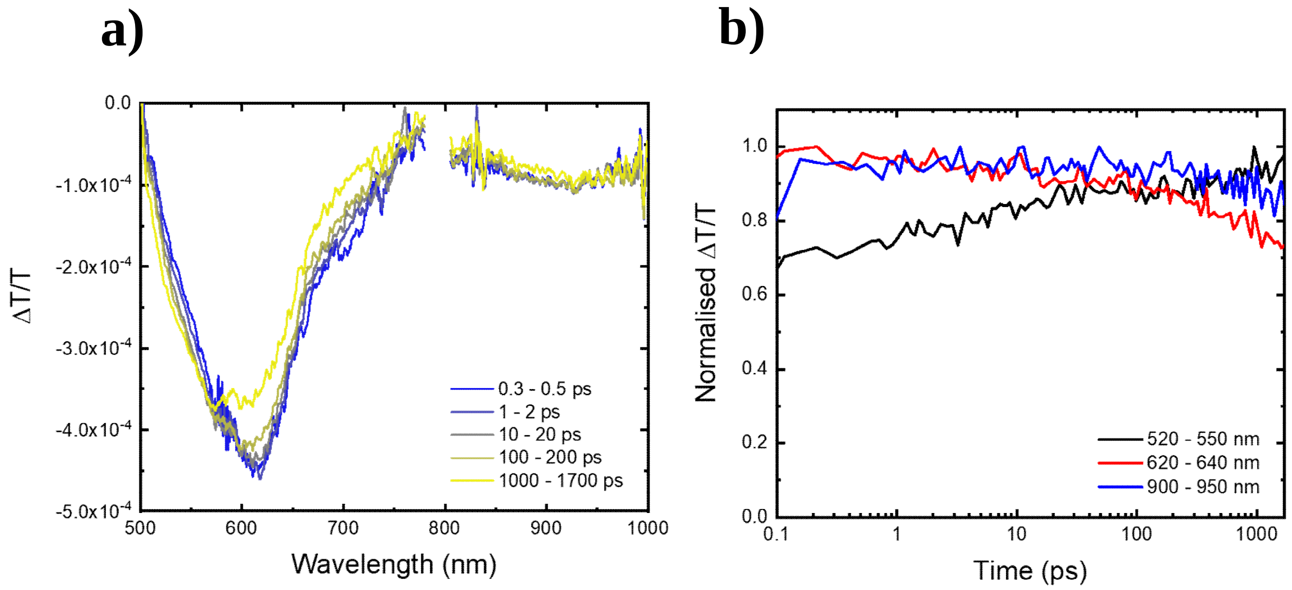

Supplementary Figure 5. Short-time TA of TTM-3PCz:CBP with 600 nm excitation. a) Δ*T/T* timeslices of TTM-3PCz:CBP (0.03:0.97) films with 600 nm excitation at 131.3 μJ/cm^2 fluence, from 0.3-0.5 ps to 1000-1700 ps. b) Normalised Δ*T/T* kinetic profiles of 520-550 nm; 620-640 nm and 900-950 nm that are photoinduced absorption (PIA) signals we assign to excited states of TTM-3PCz. Discontinuities in timeslice spectral profiles for a) are due to multiple experiments used to cover the studied wavelength probe regions.

TA of 4CzIPN:TTM-3PCz:CBP

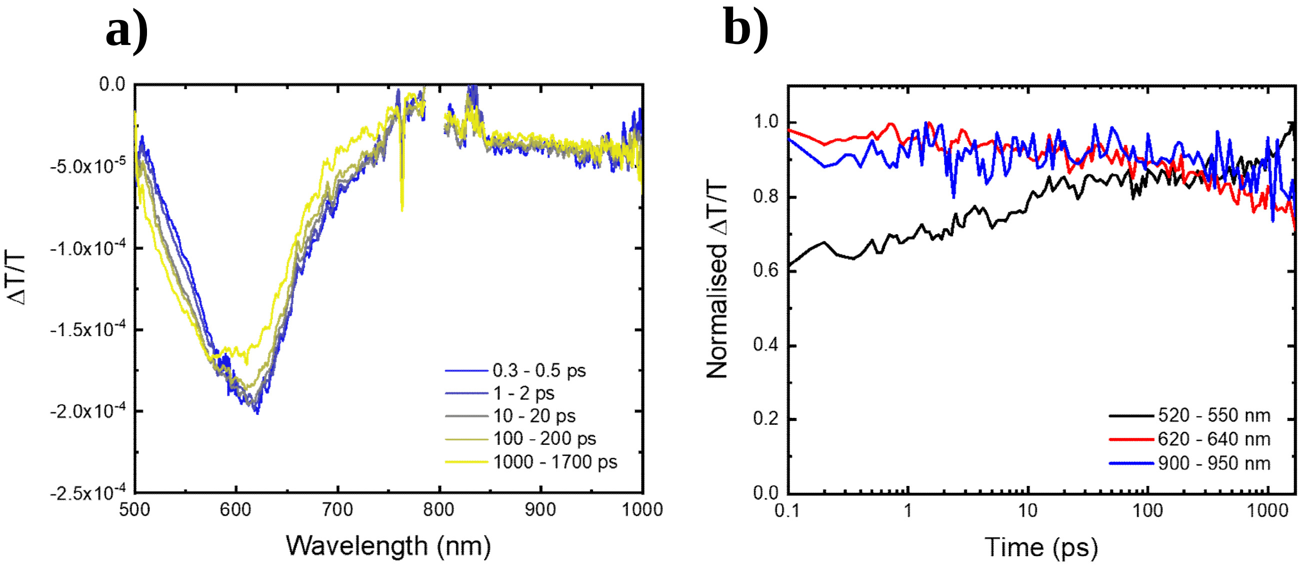

Supplementary Figure 6. Short-time TA of 4CzIPN:TTM-3PCz:CBP with 600 nm excitation. a) Δ*T/T* timeslices of 4CzIPN:TTM-3PCz:CBP (0.25:0.03:0.72) films with 600 nm excitation at 131.3 μJ/cm^2 fluence, from 0.3-0.5 ps to 1000-1700 ps. b) Normalised Δ*T/T* kinetic profiles of 520-550 nm; 620-640 nm and 900-950 nm that are photoinduced absorption (PIA) signals we assign to excited states of TTM-3PCz. Comparison of TA for 4CzIPN:TTM-3PCz:CBP and TTM-3PCz:CBP films with 600 nm excitation (Fig. S5 and S6) shows that D_1 excitons formed for TTM-3PCz does not interact with 4CzIPN. Discontinuities in timeslice spectral profiles for a) are due to multiple experiments used to cover the studied wavelength probe regions.

3. Electroluminescence time dependence of 4CzIPN:TTM-3PCz:CBP OLEDs

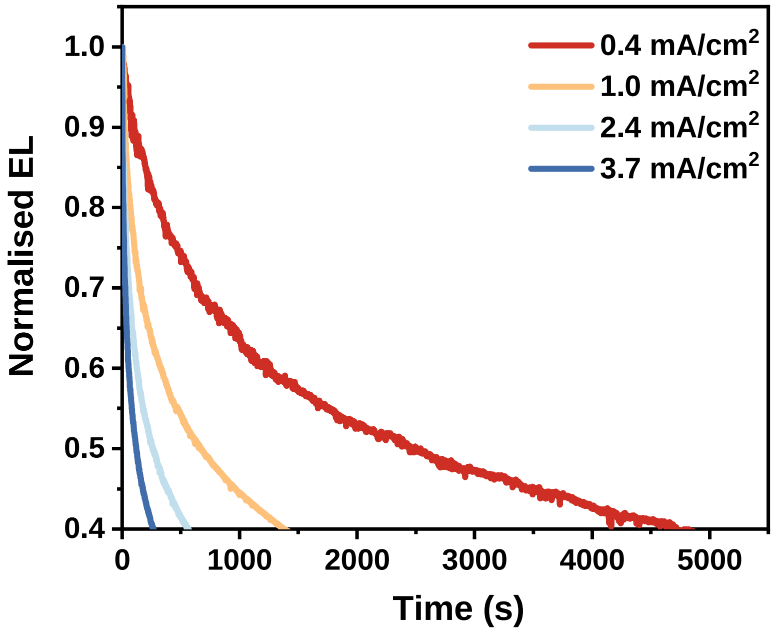

Supplementary Figure 7. Normalised EL time dependence of 4CzIPN:TTM-3PCz:CBP OLEDs with fixed current density values. The device half-time T50 values were found to be: 42 min at 0.4 mA/cm^2; 10.5 min at 1.0 mA/cm^2; 4.5 min at 2.4 mA/cm^2; 2 min at 3.7 mA/cm^2 (T50 = time for luminance to fall to half of initial value under constant current density operation).

4. Steady-state photoluminescence of 4CzIPN:TTM-3PCz:CBP film

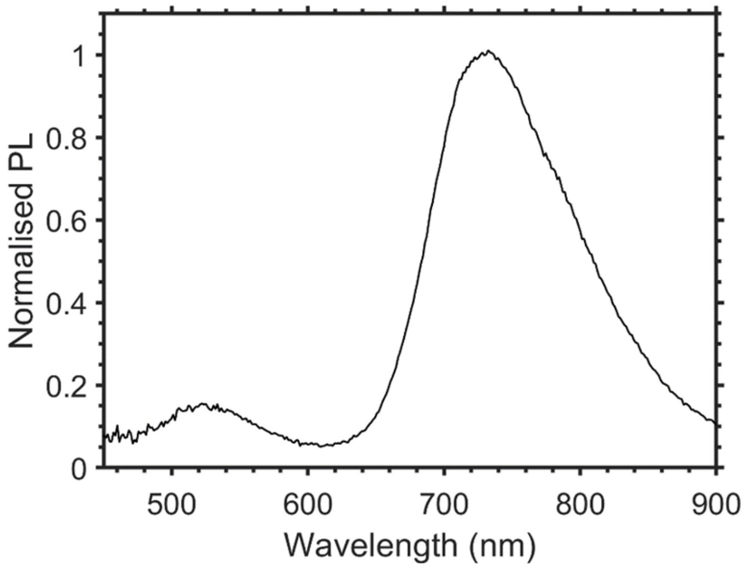

Supplementary Figure 8. Photoluminescence of 4CzIPN:TTM-3PCz:CBP film. Steady-state PL of 4CzIPN:TTM-3PCz:CBP (0.25:0.03:0.72) from 405 nm excitation.

5. Magneto-conductance studies of 4CzIPN:CBP and 4CzIPN:TTM-3PCz:CBP OLEDs

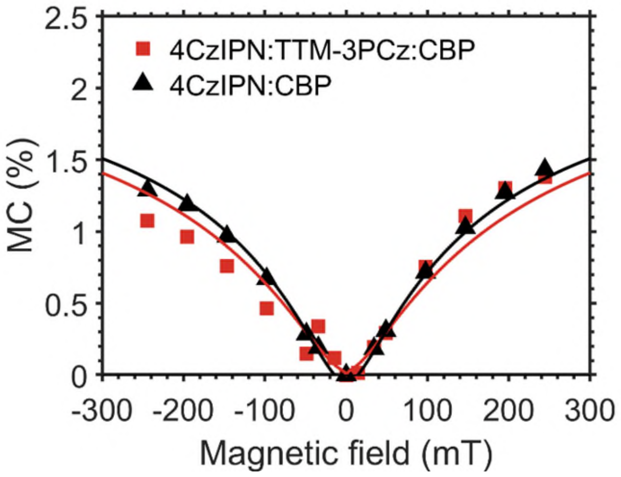

Supplementary Figure 9. Magneto-conductance (MC) studies of 4CzIPN:CBP, 4CzIPN:TTM-3PCz:CBP OLEDs. Studied OLEDs were fabricated according to Fig. 4a, main text with emissive layers (30 nm): 4CzIPN:TTM-3PCz:CBP (0.25:0.03:0.72), red squares; 4CzIPN:CBP (0.25:0.75), black triangles. Data collected simultaneously with magneto-electroluminescence from Fig. 4f.

6. Temperature dependence on transient photoluminescence of 4CzIPN:CBP and 4CzIPN:TTM-3PCz:CBP films

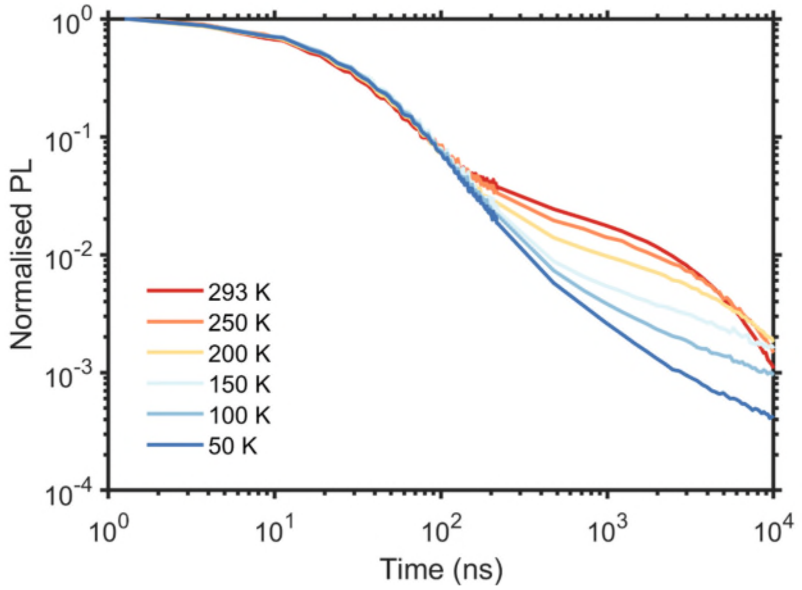

Supplementary Figure 10. Temperature dependence of transient photoluminescence profiles for 4CzIPN:CBP film for 4CzIPN emission. Transient PL profiles for nano-to-micro-second time ranges (with 400 nm excitation at 5 μJ/cm^2) of 4CzIPN:CBP (0.25:0.75) at various temperatures from 50 to 293 K, averaged for total 4CzIPN emission (450-650 nm range).

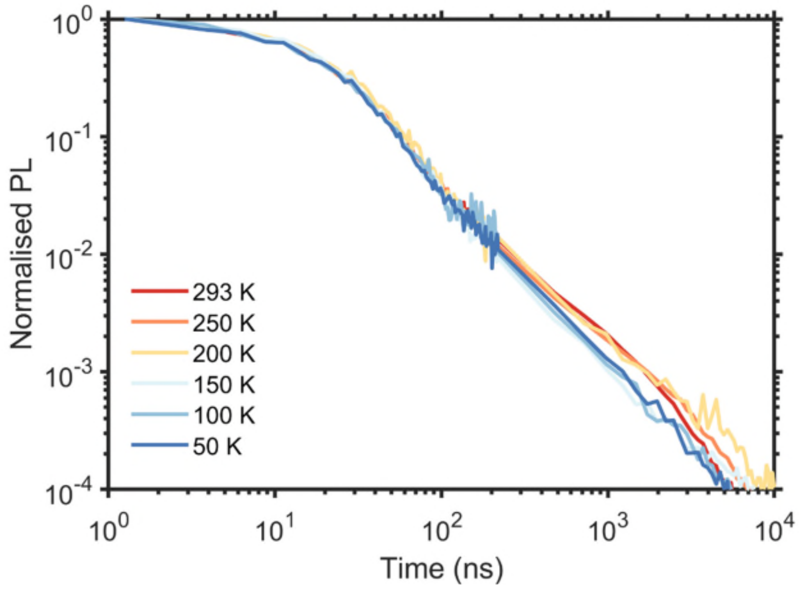

Supplementary Figure 11. Temperature dependence of transient photoluminescence profiles for 4CzIPN:TTM-3PCz:CBP film for TTM-3PCz emission. Transient PL profiles for nano-to-micro-second time ranges (with 400 nm excitation at 5 μJ/cm^2) of 4CzIPN:TTM-3PCz:CBP (0.25:0.03:0.72) at various temperatures from 50 to 293 K, averaged for TTM-3PCz emission (700-800 nm range).
